# Temporal and Sex-Related Differences in Knee Biomechanics Over the Course of the Varsity Athletic Season: Pre- and Postseason Knee Kinematics in Collegiate Varsity Athletes Using Kinect

**DOI:** 10.1177/23259671251386445

**Published:** 2025-11-07

**Authors:** Tatiana Joseph, Athanasios Babouras, Kevin Yan Zhao, Jason Corban, Paul A. Martineau

**Affiliations:** † Faculté de Médecine, Université de Montréal, Montreal, Canada; ‡ Department of Experimental Surgery, McGill University, Montreal, Canada; § Faculty of Medicine, McGill University, Montreal, Canada; ‖ Division of Orthopaedic Surgery, McGill University Health Centre, Montreal, Canada; ¶ Department of Electrical and Computer Engineering, Concordia University, Montreal, Canada; # Department of Health, Kinesiology and Applied Physiology, Concordia University, Montreal, Canada; ** Research Institute of the McGill University Health Centre, Montreal, Canada; Investigation performed at Research Institute of the McGill University Health Centre, Montreal, Canada

**Keywords:** anterior cruciate ligament, injury prevention, drop vertical jump, Microsoft Kinect

## Abstract

**Background:**

Anterior cruciate ligament (ACL) tears can be a source of significant morbidity, with the potential for career-altering implications for athletes who sustain them. Specific knee biomechanics during a drop vertical jump have been shown to be associated with an increased risk for ACL injury in collegiate varsity athletes. Presently, the evolution of these kinematics from preseason to postseason is not well-understood.

**Purpose:**

To compare preseason and postseason knee biomechanics during a drop vertical jump in collegiate varsity athletes and identify changes in ACL injury risk.

**Study Design:**

Cohort study; Level of evidence, 2.

**Methods:**

A total of 114 collegiate athletes were prospectively enrolled. Of these 114, 67 athletes (male, 21 [31%]; female, 46 [69%]) completed properly captured preseason and postseason drop vertical jumps tracked by an affordable motion capture system. Initial coronal (IC), peak coronal (PC), and peak sagittal (PS) angles of the knee were compared between preseason and postseason using the Wilcoxon signed-rank test and paired-samples *t* test. Athletes at high risk for ACL injury were identified based on published cutoff angles: IC angle >2.96°, PC angle >6.16°, and PS angle <93.82°, then the distribution of these athletes was compared.

**Results:**

In male athletes, all preseason knee angles were in the low-risk range. At postseason, men presented a nonsignificant reduction in mean IC and PC knee angles and a nonsignificant reduction in mean PS angle (90.88 ± 10.69). On average, female athletes were at high risk at preseason according to mean IC and PS angles (4.24 ± 1.09 and 92.90 ± 6.94, respectively). There was a statistically significant reduction in mean IC angle (mean difference [MD], 2.23; *P* = .03) and mean PC angle (MD, 0.76; *P* = .04); however, mean IC angle remained in the high-risk range. There was a nonsignificant reduction in mean PS angle, which remained within the high-risk range (MD, 3.96; *P* = .24).

**Conclusion:**

Our study demonstrated that female collegiate varsity athletes demonstrate higher risk knee biomechanics in comparison with their male counterparts. Even with improved biomechanics as their season advances, female athletes have a persistently low PS angle, leaving them at high risk of ACL injury. Using a portable and reliable motion capture system may facilitate monitoring knee kinematics, which could translate into a tool for ACL injury prevention in athletes.

Noncontact anterior cruciate ligament (ACL) injuries, which account for 70% of all ACL injuries in both male and female athletes, commonly occur during planting and cutting as well as landing maneuvers.^11,13^ Most often, the injury occurs when athletes are decelerating (eg, when changing directions or landing from a jump). Sports such as rugby, handball, and American football have been shown to have a higher incidence of ACL injury.^7^ These injuries can be a potential source of significant morbidity for athletes who sustain them while also imposing a significant financial burden on patients, teams, and health care systems.^18^

The rate of ACL injuries is highest during high school years for both sexes but is higher in female athletes.^3^ The overall incidence diminishes with increasing age; however, women tend to represent a larger proportion of injured athletes. For example, Prodromos demonstrated a roughly 3 times higher incidence of ACL tears among women in soccer and basketball than among male players.^20^ They also stated that with all sports considered, women are 8 to 9 times more likely to sustain an ACL injury than their male counterparts.^13,20^ The reasons for such disparity are multifactorial and include risk factors for noncontact ACL injuries that can be categorized as intrinsic (sex, biomechanical, knee anatomy, and hormonal) and extrinsic (environmental).^2^

Traditional 3-dimensional motion analysis laboratories are effective for tracking knee kinematics; however, they tend to be expensive and inaccessible for routine screening. For screening purposes, alternative motion tracking devices that are portable and cost-effective, yet capable of providing accurate measurements, are desired. In our laboratory, an affordable motion capture system (Kinect; Microsoft Corp) has been shown to produce intraclass correlation coefficient (ICC) values ranging from 0.771 to 0.917 for assessing drop vertical jump (DVJ) parameters when compared with the current gold standard Vicon system.^4^ For reference, ICC values >0.75 have been demonstrated to indicate excellent interrater reliability for kinematic assessment.^10^

Previous research has investigated specific DVJ parameters that are associated with increased risk for ACL injury. According to Corban et al,^4^ knee angles, obtained during preseason physicals, that correlate with ACL injuries include increased initial coronal (IC) angle, increased peak coronal (PC) angle, and decreased peak sagittal (PS) angle during a DVJ. Based on receiver operating characteristic curve analysis, IC angle showed good prognostic ability, whereas the prognostic ability of the PC and PS angles was excellent.^4^ The cutoff angles identified in this investigation were IC angle > 2.96°, PC > 6.16°, and PS < 93.82°.

However, despite being a potentially effective adjunct for determining at-risk athletes during preseason evaluation, it is unclear how these knee biomechanics, and the associated risks for ACL injury, change in athletes over the course of their season. There is evidence to suggest that there is temporal variation in the rates of ACL injury when comparing preseason activity to early “regular” season play.^1^ As such, the goal of the present study was to compare preseason and postseason knee biomechanics for changes and identify correlation with ACL injury risk in collegiate varsity athletes. We hypothesized that there is a significant difference in knee biomechanics in all athletes when comparing preseason and postseason kinematics, especially in terms of knee flexion and valgus.

## Methods

### Ethical Approval

This investigation was a prospective cohort study. Ethics approval was received from the research ethics office of the Faculty of Medicine and Health Sciences of McGill University before the start of this investigation, and informed consent was obtained from all participants in this study. After consultation with the coaches, training staff, and medical staff, all participants were tested during predetermined times. The enrollment period lasted from August 2022 to January 2023 inclusively.

### Participants and Injury Surveillance

Collegiate athletes were enrolled prospectively before the start of their athletic season. The preseason testing occurred during scheduled preseason physical examinations between August and September 2022, while the postseason testing occurred at predetermined times throughout the months of December 2022 to January 2023. Exclusion criteria were athletes <18 years or >30 years or the presence of a lower limb injury at the time of consent. Data collection included sex, age, body mass index, and sport played. Athletes were followed throughout the season for noncontact ACL injury. Suspected ACL injuries were confirmed by magnetic resonance imaging and, for those undergoing ACL reconstruction, under direct visualization with arthroscopy.

### Motion Capture System

The motion capture system we developed uses the Kinect V2 (Microsoft Corp) and its body tracking framework to track a participant's joints in real time and store the information of 25 joints for each recording frame in a JSON-formatted text file. Each joint has an *X*, *Y*, and *Z* value corresponding to its position in 3-dimensional space. The Kinect V2 was mounted on a tripod 1.08 m high and 2.5 m away from the athletes, who were instructed to begin their DVJ from a 31-cm box. Participants were instructed to stand with the balls of their feet over the edge of the box while facing the camera, let their weight fall forward until they fell off the box, land with both feet simultaneously, absorb their weight, and immediately bounce back up vertically to jump as high as possible. Participants were offered 1 practice DVJ to ensure the jump would be performed properly. Then, 3 DVJs were performed in succession. The joint information was loaded and run through custom algorithms, which extracted the key moments of the jump and stored the desired knee angles. The results were then exported in CSV format for analysis. The system has been validated and published in the *American Journal of Sports Medicine*.^4^

### Statistical Analysis

**Figure 1.**
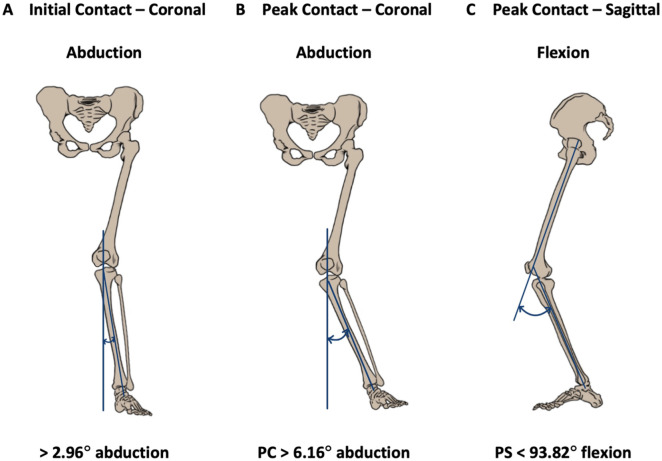
Images of the optimal knee joint kinematic angle cutoffs associated with increased risk of noncontact anterior cruciate ligament tear as generated by the motion capture system software. (A) Initial coronal angle >2.96°. (B) Peak coronal (PC) angle >6.16°. (C) Peak sagittal (PS) flexion angle <93.82°.

Each participant performed 3 DVJs. The first testing was conducted during the varsity athletes’ respective preseason physical sessions. The second testing was conducted at the respective postseason session organized singularly for our testing. For each jump, IC, PC, and PS were captured (Figure 1). “Faulty” angles were defined as knee angles measured by the motion capture system that were not physically feasible and were inconsistent with the visual recordings. Angles flagged as faulty data were manually reviewed by 2 authors (T.J. and A.B.) and then deemed acceptable or not. Data were collected for both knees for all 3 DVJs per athlete.

All statistical tests were performed using SPSS Version 27 (IBM), and statistical significance was set at *P* < .05. The normality of each parameter was determined using the Shapiro-Wilk test. IC, PC, and PS angles were compared between preseason and postseason time points in all athletes, female athletes, male athletes, and by individual varsity sport. The Wilcoxon signed-rank test was used to compare nonparametric parameters, and the paired-samples *t* test was used to compare parametric parameters.

Subsequently, preseason and postseason DVJ parameters were respectively compared between female and male athletes. Nonparametric parameters were compared using the Mann-Whitney *U* test while parametric parameters were compared using the independent-samples *t* test.

A post hoc power analysis demonstrated that a sample size of 16 pairs would be required to detect a difference of 0.80° in IC and PC angles assuming a standard deviation of differences of 1.00°, and a difference of 8.00° in PS angles assuming a standard deviation of differences to be 10.00°, with 80% power at 5% level of significance.^6^

## Results

**Figure 2.**
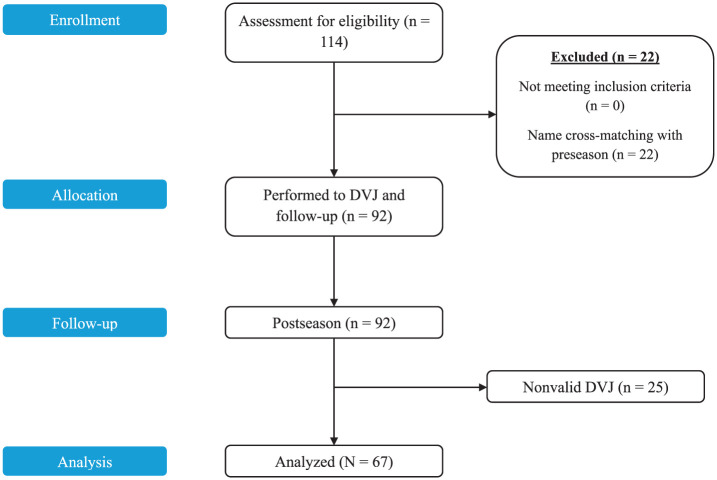
STROBE (Strengthening the Reporting of Observational Studies in Epidemiology) flowchart. DVJ, drop vertical jump.

A total of 114 athletes were recruited for the postseason testing. Of these, 22 athletes were excluded because they had not participated in the preseason analysis. Of the 92 remaining, 25 had to be dismissed because of nonvalid DVJ, due to poor execution or poorly recorded angles. The remaining 67 participants comprised 46 women and 21 men (Figure 2, Table 1). These varsity athletes were from 5 different sports, including basketball, rugby, soccer, volleyball, and football (Table 1). The measured knee angles (IC, PC, PS) at preseason and postseason are summarized in Table 3. All sports started their season in late August, and the end of their respective seasons varied. The time interval between the pre- and postseason testing for each sport is shown in Table 2.

**Table 1:** Demographic Information of Participants With Valid Drop Vertical Jumps (N = 67)*^a^*

*Characteristics*	Male Athletes	Female Athletes	All Athletes
*Participants*	21	46	67
*Age*, *y*	19.67 ± 1.88	20 ± 1.94	20 ± 1.95
*Height*, m	1.81 ± 0.08	1.73 ± 0.08	1.76 ± 0.09
*Weight*, *kg*	79.19 ± 12.30	68.23 ± 9.62	71.67 ± 11.63
*Body mass index*, *kg/m*^*2*^	23.93 ± 3.26	22.52 ± 2.31	22.96 ± 2.70
*Previous noncontact knee injuries*	1	4	5
Varsity Sport Teams			
*Soccer*	12	8	20
*Volleyball*	0	19	19
*Rugby*	8	9	17
*Basketball*	0	10	10
*Football*	1	0	1

a Data are presented as mean ± SD or n.

**Table 2:** Time Interval Between Preseason and Postseason Testing and Timing of Postseason Testing Relative to Competitive Season*^a^*

*Sport*	Test Date, Preseason	Test Date, Postseason	Date of Season End	Interval Between End of Season and Postseason, d
*Soccer* *M*	August 15, 2022	December 5, 2023	October 10, 2022	56
*F*	August 15, 2022	January 17, 2023	October 28, 2022	81
*Volleyball* *F*	September 1, 2022	January 24, 2023	December 12, 2022	43
*Rugby* *M*	August 1, 2022	January 17, 2023	November 4, 2022	74
*F*	August 26, 2022	January 13, 2023	October 23, 2022	82
*Basketball* *F*	September 1, 2022	January 24, 2023	December 3, 2022	52
*Football* *M*	August 11, 2022	January 17, 2023	October 29, 2022	80
Mean ± SD				67 ± 16

a Data are presented as n unless otherwise indicated. F, female; M, male.

**Table 3:** Male and Female Athletes With Safe and At-Risk Angles*^a^*

Sex	IC Safe	IC at Risk	PC Safe	PC at Risk	PS Safe	PS at Risk
Preseason						
Male	12	9	20	1	16	5
Female	9	37	29	17	24	22
Postseason						
Male	17	4	21	0	10	11
Female	18	28	35	11	16	30

a Data are presented as n. IC, initial coronal angle; PC, peak coronal angle; PS, peak sagittal angle.

### Preseason Results

**Figure 3.**
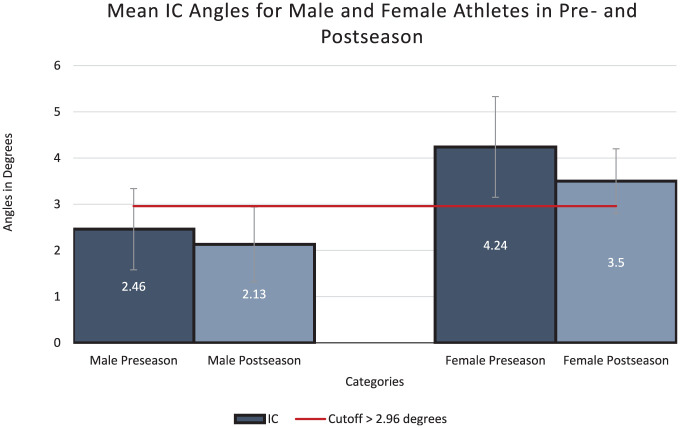
The comparison between both men and women for initial coronal (IC) angles in preseason and postseason along with their respective *P* value. All data shown are in degrees.

**Figure 4.**
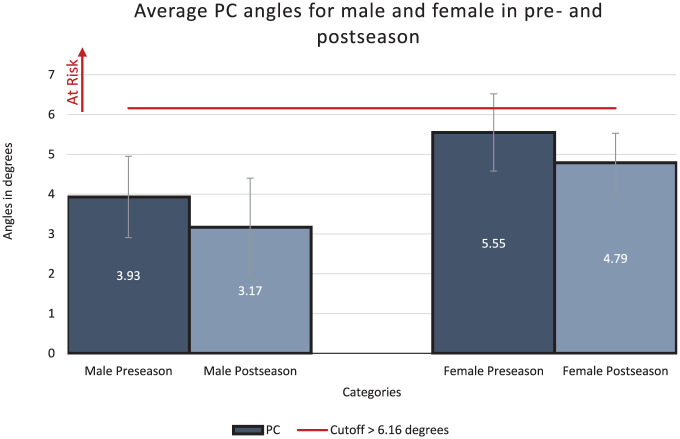
The comparison between both men and women for peak coronal (PC) angles in preseason and postseason along with their respective *P* value. All data shown are in degrees.

**Figure 5.**
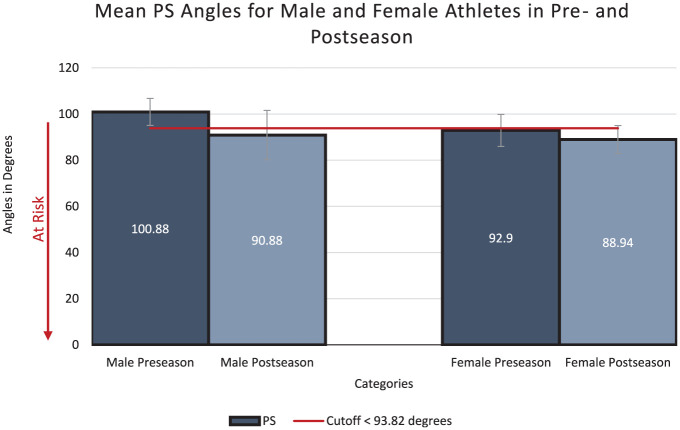
The comparison between both men and women for peak sagittal (PS) angles in preseason and postseason along with their respective *P* value. All data shown are in degrees.

At preseason, the mean IC (2.46° ± 0.88°), PC (3.93° ± 1.02°), and PS (100.88° ± 5.85°) angles for male athletes were below the threshold associated with increased risk of ACL injury (Figures 3-5). For female athletes, the mean values for 2 (IC and PS) out of 3 angles were within the high-risk range,^4^ putting them at higher overall risk for ACL injury (IC, 4.24° ± 1.09°; PC, 5.55° ± 0.97°; PS, 92.90° ± 6.94°). Female athletes also had significantly greater IC and PC values, and significantly smaller PS angles when compared with male athletes, indicating greater risk for ACL injury (Figures 3-5). Overall, 37 out of 46 women presented a high-risk IC angle, and 22 out of 46 women presented a high-risk PS angle (Table 3).

### Postseason Results

At postseason follow-up, male athletes had improved IC and PC angles (IC, 2.13° ± 0.81°; PC, 3.17° ± 1.23°) but worse PS angles (90.88° ± 10.69°) (Figures 3-5). The mean IC and PC angles remained below the high-risk cutoffs, while the mean PS angle was within the high-risk range. Similarly, female athletes also presented improved IC and PC, but worse PS, angles (IC, 3.50° ± 0.70°; PC, 4.79° ± 0.74°; PS, 88.94° ± 6.05°) (Figures 3-5). Although the IC angle improved, it remained above the high-risk cutoff for ACL injuries, while the PC angle was below the cutoff for increased risk of ACL injury.^4^ As the mean PS angle decreased, it continued to remain in the high-risk range. Overall, 11 out of 21 men presented a high-risk PS angle, while 28 out of 46 women presented a high-risk IC angle and 30 out of 46 women presented a high-risk PS angle (Table 3).

### Differences Between Preseason and Postseason DVJ Knee Angles

Male athletes presented no statistically significant changes between their preseason and postseason jumps for IC (mean difference [MD], −0.33°; *P* = .57), PC (MD, −0.76°; *P* = .07) and PS angles (MD, −1.93°, *P* = .05) (Table 4). In female athletes, there was a statistically significant reduction in IC angle (MD, –2.23°; *P* = .03) and PC angle (MD, −0.76°; *P* = .04), while PS angle was not significantly different (MD, –3.96°; *P* = .24) (Table 4).

**Table 4:** Comparison Between Men and Women for Mean IC, PC, and PS Angle*^a^*

Sex	Mean IC	*P*	Mean PC	*P*	Mean PS	*P*
Preseason						
Male vs female	1.78	.007	1.62	.002	7.99	.04
Postseason						
Male vs female	–3.12	.002	–0.31	.03	–0.65	.52
Preseason vs postseason						
Male	–0.33	.57	–0.76	.07	–10.00	.05
Female	–2.23	.03	–0.76	.04	–3.96	.24
Range of error	0.916		0.897		0.776	

a Data are presented in degrees. IC, initial coronal angle; PC, peak coronal angle; PS, peak sagittal angle.

### ACL Injury Status

Two out of 67 athletes (2.99%) had confirmed ACL injury; 1 in soccer and 1 in rugby. Both athletes were female. The diagnosis was made through primary assessment of physical therapist or athletic therapist on the field. This was followed up with an assessment by a physician, and the diagnosis was confirmed by magnetic resonance imaging. The soccer player had preseason angles of the following: IC, 4.53° ± 0.50°; PC, 6.67° ± 0.16°; and PS, 91.69° ± 3.88°; the rugby player had the following angles: IC, 3.88° ± 0.43°; PC, 5.01° ± 0.69°; and PS, 119.8° ± 2.60°.

## Discussion

The major findings of this study demonstrated that male athletes’ knee angles were in the low-risk range during pre- and postseason. They presented a nonsignificant reduction in mean IC and PC knee angles and a nonsignificant reduction in mean PS angle (90.88 ± 10.69) compared with their preseason mean angles. Female athletes were at high risk at preseason according to mean IC and PS angles (4.24 ± 1.09 and 92.90 ± 6.94). There was a statistically significant reduction in mean IC angle (2.23; *P* = .03) and mean PC angle (0.76; *P* = .04); however, mean IC angle remained in the high-risk range. There was a nonsignificant reduction in mean PS angle which remained within the high-risk range (3.96; *P* = .24).

In the present study, we investigated intrinsic biomechanical risk factors, specifically the IC, PC, and PS knee flexion angles and examined how they differed between sex and changed over the course of the athletic season. With regard to sex-related differences, we found a statistically significant difference in IC, PC, and PS angles between male and female athletes during preseason testing (Table 4). Female athletes in this cohort also demonstrated high-risk angles for IC and PS during the preseason, which remained high risk in the postseason (Table 3). Male athletes continued to have low-risk values during postseason testing (Table 3). Despite the fact that collegiate varsity athletes are likely at the peak of their physical form at preseason, these results indicate the presence of intrinsic biomechanical differences between male and female athletes that are known to be associated with increased risk for noncontact ACL injury. These findings suggest biomechanical evidence to support widely documented demographic data showing that women are at increased risk of noncontact ACL injury, while also highlighting the plasticity of knee biomechanics, which may deteriorate over the course of the athletic season in a sex-specific manner.

Noncontact female ACL injuries are most commonly caused during planting and cutting (29%), straight knee landing (28%), or 1-step stop landing with the knee hyperextended (26%).^9^ Biomechanics, muscle strength, and physiognomy make them more prone to these injuries. Further, it has been shown that ACL tears occur with muscle fatigue.^16^ Muscles such as the quadriceps, hamstrings, gastrocnemius, soleus, and tibialis anterior are necessary to stabilize the knee. These muscles are associated with movements of valgus in the coronal plane and knee flexion in the sagittal plane. Increased valgus and decreased flexion have been shown to increase the risk of ACL tears.^9,12^ Women were also shown to have larger PC and smaller PS angles than men, which may increase their chances of injury.^15^ It was reported that “women tend to have less knee flexion angles, more knee valgus angles, greater quadriceps activation, and lower hamstring activation in comparison to men.”^15^ Similarly, in the present study, female athletes demonstrated significantly greater IC and PC angles, indicating more knee valgus, and smaller PS angles, indicating less knee flexion, during their preseason DVJ jumps when compared with the male athletes.

Cortes et al^5^ investigated the relationship between knee kinematics in relation to fatigue in a cohort of Division I collegiate soccer athletes. During landing tasks, they found that fatigue decreased knee flexion angle. In female football players, maximal knee flexion angle has been shown to be reduced immediately postmatch and was still reduced 48 hours after the match.^17^ We surmise that in progressing from preseason to postseason, cumulative fatigue helps to explain the results of this investigation, particularly within the female cohort, even though not explicitly measured.

In this study, the average male athlete started the season with mean IC, PC, and PS angles that were all low risk based on published cutoffs. IC and PC angles further decreased, indicating less knee valgus, in a nonsignificant manner at postseason. Overall, male athletes were considered to have stable biomechanics related to their coronal knee angles when comparing between preseason and postseason. While PS angles decreased from preseason to postseason, indicating less knee flexion and hypothetically greater risk for ACL injury, the difference was not statistically significant. Further, the mean postseason PS angle (92.90° ± 6.94°) was close to the published cutoff value (93.82°) with a standard deviation that exceeded the difference between the 2 values. Men have been shown to be able to compensate for knee instability by increasing their power, strength, and coordination more than women, which, based on the results of this investigation, may partially explain the increased risk for ACL tears in women.^9^

In contrast, the average female athlete started the season with less-protective biomechanics than their male counterparts, with high-risk mean IC and PS angles. While the mean IC angle significantly improved at postseason, indicating improved biomechanics as the season progressed, the postseason mean IC angle remained within the high-risk range. There was a nonsignificant reduction in PS angle at postseason, indicating less knee flexion and elevated risk for ACL injury. Of note, the changes in IC and PS angles were outside of the measurement error of the motion capture system. Overall, this correlated with the documented ACL injuries of the 2022 to 2023 varsity season: both athletes who suffered ACL injuries were female (2 athletes out of 67: rugby and soccer). Therefore, a persistence and possible further worsening of suboptimal knee flexion in female athletes, potentially exacerbated by muscle fatigue, may contribute to the increased odds of ACL injury in comparison with male athletes.

### Prevention Programs

During the season, knee biomechanics were measured and shown to remain within the high-risk range for female athletes. With this information, the possibility of using Kinect to track knee biomechanics throughout a season and identify at-risk athletes could become a significant tool for ACL prevention. With this information, the possibility of designing integrated plyometrics, strengthening, balance, endurance, and neuromuscular training, such as the warm-up program (FIFA 11+^8^) could be considered. The use of targeted programs may help reduce the risk of ACL injury in female athletes by up to 90%.^21^ The use of screening tests, then, becomes essential to identify high-risk athletes. For example, the FIFA 11+ warm-up program, designed by FIFA Medical Assessment and Research Centre, has been shown to reduce sports injuries by up to one-third of young female athletes.^19,21^ More specifically, preseason neuromuscular and proprioception programs designed to avoid “at-risk positions” during landing has been shown to reduce the risk of ACL injury by 1.83 times.^21^ Ultimately, maintenance of sufficient knee flexion is valuable in female athletes to address the biomechanical deficiencies observed in the present study.

## Limitations

A potential limitation of this study is the grouping of data from athletes participating in a variety of different sports. This could potentially mask the results of certain sports that are considered to be higher risk for noncontact injury compared with other sports. However, care was taken to select sports in which planting-and-cutting maneuvers were performed. Further, we hypothesized that training regimens, intensity, and sport requirements were similar across sports and between sexes. Furthermore, current research suggests that demands during a game are similar for men and women. Each varsity program also imposes a strict training regimen of field practices, conditioning, and strength training. Another limitation of the present study is that it did not incorporate detailed data (number of games, hours of training on and off the field) for each team to precisely compare the physical demands required. The information on hours of training, number of games, and recuperation postseason was not available and could have helped to quantify variables that related to exertion and fatigue. Finally, the postseason jumps were all recorded within 3 months of the end of the athletes’ respective seasons. Therefore, the period of recuperation postseason may have varied between teams. We currently have no information available regarding training during these 3 months. Variations in recovery and injury prevention training could have influenced the measured angles, as knee biomechanics immediately at the end of the season were not assessed. Athletes tested shortly after the season may still have experienced residual fatigue, potentially leading to altered movement patterns and joint mechanics, whereas those tested later may have regained strength and stability, affecting the measured angles. The need for a standardized postseason follow-up would be needed in the future.

In terms of kinematic assessment, our Kinect V2 only captures at 30 Hz, which may have decreased granularity compared with traditional motion analysis systems. However, our system has been validated against the gold standard Vicon system and should be sufficient to perform the kinematic assessment for investigation.^14^

## Conclusion

Our study demonstrated that female collegiate varsity athletes exhibit higher risk knee biomechanics in comparison with their male counterparts. Even with improved biomechanics as their season advances, female athletes have a persistently low PS angle, leaving them at high risk of ACL injury. Using a portable and reliable motion capture system may facilitate the monitoring of knee kinematics, which could translate into a tool for ACL injury prevention in athletes.
